# Evaluation of linkage disequilibrium, population structure, and genetic diversity in the U.S. peanut mini core collection

**DOI:** 10.1186/s12864-019-5824-9

**Published:** 2019-06-11

**Authors:** Paul I. Otyama, Andrew Wilkey, Roshan Kulkarni, Teshale Assefa, Ye Chu, Josh Clevenger, Dan J. O’Connor, Graeme C. Wright, Stanley W. Dezern, Gregory E. MacDonald, Noelle L. Anglin, Ethalinda K. S. Cannon, Peggy Ozias-Akins, Steven B. Cannon

**Affiliations:** 1 Agronomy Department, Iowa State University, Ames, IA USA; 2 ORISE Fellow, Corn Insects and Crop Genetics Research Unit, USDA-ARS, Ames, IA USA; 3 Institute of Plant Breeding, Genetics, and Genomics, University of Georgia, Tifton, GA USA; 4 Mars-Wrigley Confectionery, Center for Applied Genetic Technologies, Athens, GA USA; 5 Peanut Company of Australia, Kingaroy, Qld Australia; 6 University of Florida, Gainesville, FL USA; 7 International Potato Center, Lima, Peru; 8 Computer Science Department, Iowa State University, Ames, IA USA; 9 Corn Insects and Crop Genetics Research Unit, USDA - Agricultural Research Service, 1017 Crop Genome Lab 819 Wallace Rd, Ames, IA 50011-4014 USA

**Keywords:** Linkage disequilibrium, Population structure, Phylogenetic network tree, Genetic diversity, Genome wide association

## Abstract

**Background:**

Due to the recent domestication of peanut from a single tetraploidization event, relatively little genetic diversity underlies the extensive morphological and agronomic diversity in peanut cultivars today. To broaden the genetic variation in future breeding programs, it is necessary to characterize germplasm accessions for new sources of variation and to leverage the power of genome-wide association studies (GWAS) to discover markers associated with traits of interest. We report an analysis of linkage disequilibrium (LD), population structure, and genetic diversity, and examine the ability of GWA to infer marker-trait associations in the U.S. peanut mini core collection genotyped with a 58 K SNP array.

**Results:**

LD persists over long distances in the collection, decaying to *r*^2^ = half decay distance at 3.78 Mb. Structure within the collection is best explained when separated into four or five groups (K = 4 and K = 5). At K = 4 and 5, accessions loosely clustered according to market type and subspecies, though with numerous exceptions. Out of 107 accessions, 43 clustered in correspondence to the main market type subgroup whereas 34 did not. The remaining 30 accessions had either missing taxonomic classification or were classified as mixed. Phylogenetic network analysis also clustered accessions into approximately five groups based on their genotypes, with loose correspondence to subspecies and market type. Genome wide association analysis was performed on these lines for 12 seed composition and quality traits. Significant marker associations were identified for arachidic and behenic fatty acid compositions, which despite having low bioavailability in peanut, have been reported to raise cholesterol levels in humans. Other traits such as blanchability showed consistent associations in multiple tests, with plausible candidate genes.

**Conclusions:**

Based on GWA, population structure as well as additional simulation results, we find that the primary limitations of this collection for GWAS are a small collection size, significant remaining structure/genetic similarity and long LD blocks that limit the resolution of association mapping. These results can be used to improve GWAS in peanut in future studies – for example, by increasing the size and reducing structure in the collections used for GWAS.

**Electronic supplementary material:**

The online version of this article (10.1186/s12864-019-5824-9) contains supplementary material, which is available to authorized users.

## Background

Peanut is one of the most important oilseed crops in the world, with many desirable traits: it is high-protein and nutrient-dense, is edible without preparation, and is easily grown by small-holder farmers as well as under mechanization. Peanut has a desirable lipid profile, with oil that is higher in unsaturated fatty acids than saturated fatty acids; as such, it may provide numerous health benefits, including lowering LDL cholesterol [1]. Breeding for improved seed composition and quality therefore forms an integral part of most breeding and improvement programs.

Peanut belongs in the *Arachis* genus in the Fabaceae family. The genus has nine taxonomic sections based on morphological, geographical and cross-incompatibility characteristics across all of its 80 species. Most of the species are diploids (*2n = 2x = 20*) with four known aneuploids (*2n = 2x = 18*) and five tetraploids (*2n = 4x = 40*) including the cultivated form [2–4]. Cultivated peanut is broadly classified under two subspecies: *fastigiata* and *hypogaea*, depending on the presence or absence of flowers on the main axis [5]. There are four main market types of peanuts grown in the USA - Runner, Virginia, Spanish and Valencia types. Runner types are the most widely grown, primarily for processing into peanut butter. Market type classification is based on distinct seed size and flavor [6], which are difficult traits to accurately assess and distinguish. This study evaluates, in part, the genotypic basis for these phenotypic classifications.

The sequencing of the diploid progenitors of tetraploid peanut [7], (which is available through GenBank and PeanutBase [8]), has made it possible to develop robust genotyping platforms such as single nucleotide polymorphism (SNP) arrays for use in peanut breeding and genetics. The use of SNPs in peanut genomics and breeding comes with complexities associated with it being an allotetraploid with two highly similar subgenomes [7]. This makes it difficult to accurately call and predict SNP positions and distinguish true homologous SNPs (variants at a locus on one chromosome) from homeologous ones (variants at the corresponding loci of the chromosomes derived through polyploidy) [9, 10]. Although accurately calling and predicting SNP locations and positions in tetraploid peanut has been difficult [11, 12], an Affymetrix SNP array with 58,000 SNP positions has been developed and successfully deployed to study genetic diversity – for example, to examine relatedness in an ICRISAT diversity panel, and to resolve signatures of selection and tetrasomic recombination in a set of elite U.S. runner cultivars [13, 14]. Such advances have made it possible to deploy genomic approaches such as GWA to enhance and facilitate the discovery of quantitative trait loci (QTLs) and reliable markers for accelerated crop improvement.

Crop germplasm resources provide a valuable source for new allelic combinations for use in crop improvement programs. Peanut has a narrow genetic base due in part to its highly self-pollinating nature and its recent origin as a tetraploid. Peanut is believed to have been a result of a single hybridization event between two ancestor diploid species followed by a spontaneous doubling of chromosomes [7]. This difference in ploidy limits genetic exchange between tetraploid peanut and its wild diploid relatives in the *Arachis* genus. The use of a few elite breeding lines as founders in many breeding programs further compounds this problem [15, 16]. The effective incorporation of crop germplasm resources in breeding programs can remedy this by broadening the genetic base of future cultivars. The USDA germplasm collection has over 9000 *Arachis hypogaea* accessions and over 800 other *Arachis* species accessions [15, 16]. The large number of accessions makes it hard to utilize these resources in improvement programs thus, a core collection of 831 accessions was created to facilitate effective utilization and management [17]. A subset of the core was further selected to produce a mini core collection of 112 accessions, chosen to maximize genetic diversity [17, 18]. Collections such as the core and mini core sets, which have experienced several rounds of historical recombination, are more suitable for association mapping compared to F2 populations that generally have LD that extends over long distances.

Association mapping relies on markers close to the causative loci being in high LD with a QTL, but dropping off quickly with increasing distance such that only markers close to the QTL show a significant association with the phenotype of interest. The extent of LD and its decay with genetic distance are useful parameters for determining the number of markers needed to successfully map a QTL, and the resolution with which the trait can be successfully mapped. LD is population specific, and its decay in a population over time is influenced by the recombination rate between loci and the number of generations of recombination. Estimates of LD and the extent of decay with distance, for any population, will be affected by factors such as non-random mating, selection, mutation, migration or admixture, genetic drift and the effective population size [19]. The extent of LD can be estimated using statistical parameters, D’ and *r*^2^ [20], with *r*^2^ being the squared value of the correlation coefficient of the allelic states of two given polymorphic loci. The *r*^2^ parameter is the most commonly used because it gives a direct measure of the proportion of variance at the trait locus that can be predicted from the marker. At *r*^2^ < ~ 0.2, we expect LD to have been completely eroded, as it tends towards equilibrium. This is a commonly used criterion across literature, together with LD decay at *r*^2^ = half decay distance [21, 22].

Several factors besides LD affect the ability to identify marker-trait associations, including stratification, admixture, or cryptic relatedness within populations. Cryptic relatedness is the result of a close kinship relationship among otherwise unrelated individuals from a collection, and is hard to account for during association analysis [23]. Association results are confounded by false associations that arise due to the underlying structure of the population rather than a trait-associated locus [24–27]. Model-based methods of studying population stratification like FastStructure [28], are useful in inferring structure, as well as for relating the inference to known biology or genetic terms through the incorporation of additional information such as geographic location, species group etc. [29].

In this study, genomic characteristics of the U.S. peanut mini core collection were investigated, including LD, structure, and the ability to infer marker-trait associations in this collection. The objectives are: 1) to investigate the nature and extent of LD and how it relates to different chromosomes, subgenomes, minor allelic frequency (MAF) and subspecies groups; 2) to estimate genetic diversity and population structure within the U.S. peanut mini core collection and 3) to determine how these factors affect genome-wide association analysis in peanut.

## Results

One hundred seven diverse accessions from the U.S. peanut mini core collection were genotyped using a 58 K Affymetrix SNP array together with six commercial standards. A total of 13,527 highly polymorphic SNP markers were selected for downstream analyses. The collection was evaluated in the field (Citra, Florida, 2013–2015) and harvested seeds assayed for biochemical composition. To evaluate for ease of removing the seed coat from the seed (blanchability), accessions were planted across three environments (Australia 2013, U.S. 2013, Australia 2014) and harvested seeds were evaluated.

### Genome-wide distribution of SNPs

**Fig. 1:**
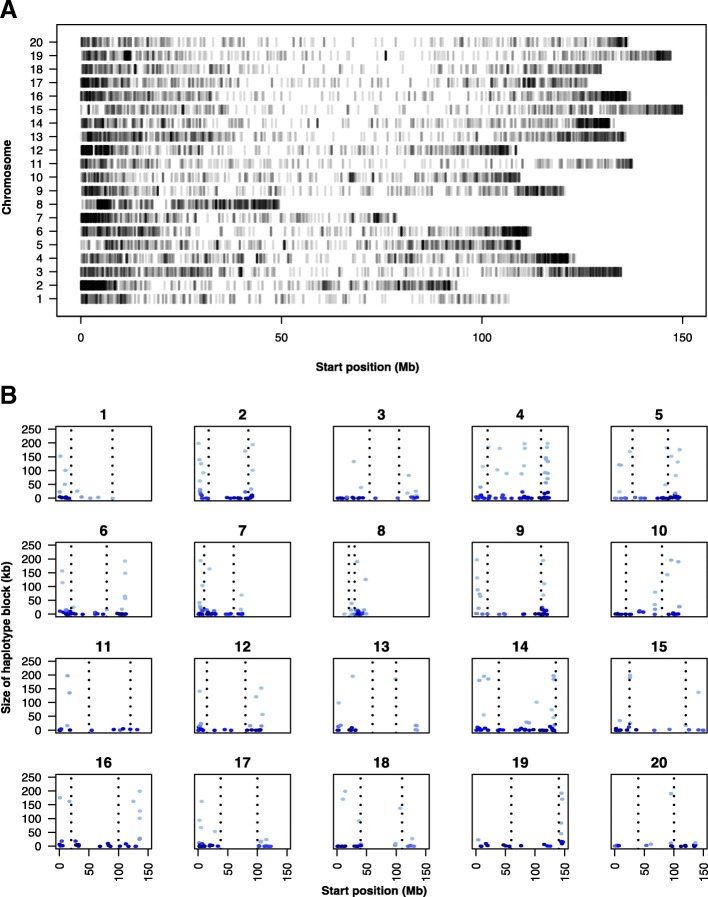
Genome-wide SNP and haplotype block distribution. **a** Distribution of SNPs identified in the minicore collection. **b** Distribution of haplotype blocks along each chromosome. Horizontal dashed lines represent pericentromeric start and end positions inferred from genetic-by-physical plots

SNP counts were approximately proportionally distributed across the 20 chromosomes. There were on average, 676 polymorphic SNPs per chromosome, and SNPs were enriched toward chromosome ends (Fig. 1a). The average distance between SNPs is 175 kb, but the SNP-to-SNP distribution is skewed: 20% of the marker-to-marker distances are less than 1 kb, and 39% are less than 10 kb. Larger regions without SNP coverage are generally in the large pericentromeric regions, where repetitive DNA makes it difficult to identify unique flanking regions around SNPs. Chromosomes 1, 10 and 11 had the lowest proportion of SNPs with 3% whereas chromosomes 2 and 12 had the highest proportion, with 8% of all polymorphic SNPs followed closely by chromosome 4 with 7% (Fig. 1a; Additional file 8: Table S2).

### Evaluation of SNP characteristics

Fourteen SNP markers, representing 0.001% of all 13,527 polymorphic markers, had high heterozygosity values ranging from 0.5 to 0.7 and of these, only three had positive inbreeding coefficients (**f**). Further, 2% (268) of the markers had heterozygosity levels ranging from 0.1 to 0.4 with average **f** of 0.8 (ranging from 0.0 to 0.9). Most of the SNPs had an inbreeding coefficient of one except for a few highly heterozygous markers that had negative inbreeding coefficients. Nine accessions showed very high levels of heterozygosity, ranging from 10 to 28% among the 13,572 selected markers (Additional file 8: Table S1). Minor allele frequency of polymorphic SNPs ranged from 0.004 to 0.5 with an estimated average of 0.13. The polymorphic information content (PIC) ranged from 0.0088 to 0.66 with an average of 0.19.

### Genetic diversity estimates

The average pairwise divergence among genotypes (π), at the 13,527 SNP locations, was 0.18799. This represents the nucleotide diversity per assayed SNP in the mini core. The expected number of polymorphic sites per nucleotide (θ), which estimates the mutation rate in the collection, was 0.18813 with 13,527 segregating sites. Tajima’s D, which estimates the normalized measure of difference between the observed (π) and expected (θ) nucleotide diversity was − 0.00252 (Additional file 8: Table S3).

### SNP haplotypes

Despite the recent evolution in peanut (hybridization followed by polyploidization), haplotype blocks identified in the mini core collection are generally not significantly larger than 200 kb in size. Depending on the sliding window size adopted, the number of haplotype blocks identified ranged from 575 blocks at 2 Mb to 590 haplotype blocks at 50 Mb window size. Increasing the sliding window size from 2 Mb to 50 Mb or larger did not have significant effect on the number of haplotype blocks identified. We note, however, that SNP densities and distributions are a limiting factor in determining both precise haplotype boundaries and GWA associations. Although the average density is relatively high compared to many older conventional marker assays (at 1 SNP per 175 kb), the density is nevertheless quite uneven, with large pericentromeric regions not covered by SNPs (the largest gaps being ~ 7 Mb in the pericentromeres of chromosomes 16 and 18). Associations in those regions may therefore aggregate the effects of several genes, and association peaks may be located at a considerable distance from a causal genomic element.

A total of 575 haplotype blocks were identified using a sliding window of 2 Mb along each chromosome. Block size varied widely, ranging from 0.002 kb to 199,905 kb with an average of 28.89 kb. The number of SNPs per block ranged from two to 32 with an average of four SNPs per block. Large haplotype blocks were mostly located within the large pericentromeric regions, which is expected since these regions have low recombination rates in general (Fig. 1b). Approximate pericentromeric start and end coordinates were inferred from genetic-by-physical plots (Additional file 8: Table S4).

### Inferring population structure and admixture proportions

**Fig. 2:**
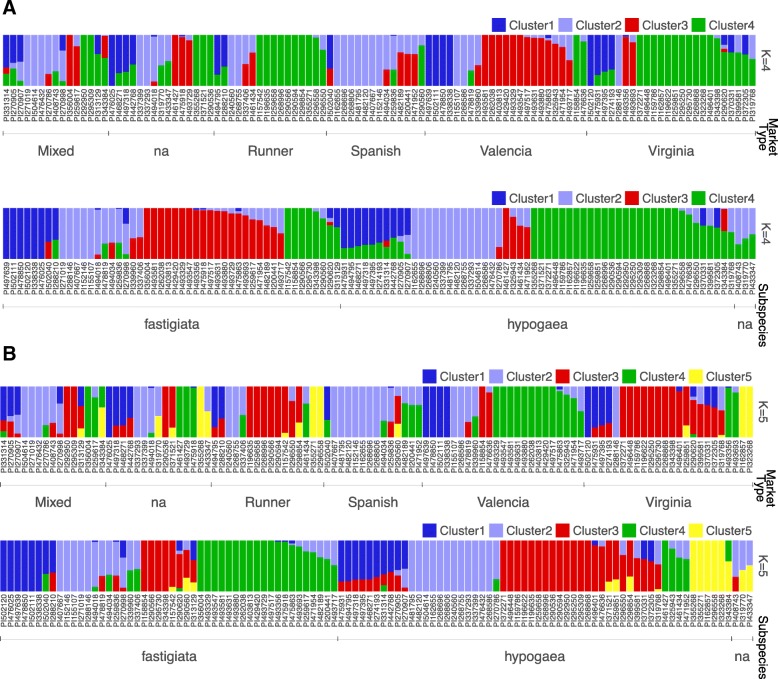
Structure within the mini core collection. Population structure plots with each vertical bar representing an accession colored according to the group to which it has been assigned. Accessions assigned to more than one group represents the extent of their admixed set of alleles. **a** structure for K = 4 subgroups ordered according to market type and subspecies (Top and Bottom bar plots respectively). **b** Population structure for K = 5 subgroups ordered according to market type and subspecies (Top and Bottom bar plots respectively)

Because inferences made on small collection sizes are particularly sensitive to minor alleles as compared to those based on larger sizes, three different MAF cutoffs were investigated. The number of genetically distinct subpopulations (K) was evaluated for each possible K ranging from 1 to 10 along with admixture levels for each accession for data filtered at MAF ≥ 0.05, ≥ 0.1 and ≥ 0.2. Subspecies, botanical variety and market type identifications [30, 31] for each genotype were included in the analysis to try and relate clustering pattern based on genetic sequences to known phenotypic classifications (Fig. 2; Additional file 1: Figure S1).

The most appropriate K used to explain structure in the data ranged from 4 to 8 depending on MAF. At MAF ≥ 0.05, K = 4 and K = 5 seems to best explain the structure in the collection. At K = 5, the 107 mini core accessions clustered approximately according to Market type – though with notable exceptions. For example, while 43 out of 107 accessions clustered in correspondence to the main market type subgroup, 34 of 107 did not correspond with the main group, and 30 accessions had either missing data or were classified as mixed. Clusters 1 and 2 had mixed membership with almost equal proportions of both subspecies whereas clusters 3 and 4 consisted predominantly of *fastigiata* and *hypogaea* subspecies respectively. The fifth cluster consisted exclusively of accessions from *hypogaea* subspecies (Fig. 2). A Fisher exact test showed significance that the observed clustering pattern at K = 5 corresponds with subspecies and market type grouping with *p*-values = 7.1 × 10^− 8^ and 7.2 × 10^− 8^ respectively.

### Phylogenetic cluster analysis

**Fig. 3:**
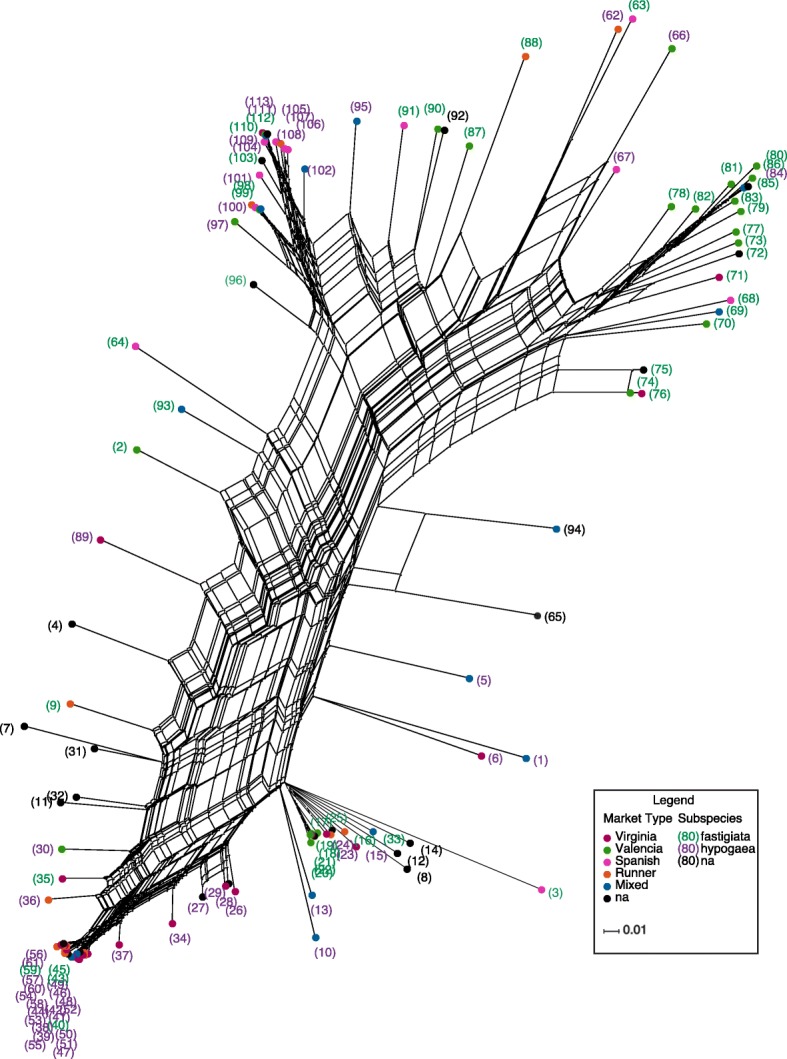
Phylogenetic network showing the relationship among accessions and cultivars. Phylogenetic network constructed using Neighbor-net based on an analysis of 6300 SNPs for all 107 minicore accessions plus 6 select cultivars used as standards in the field for a total of 113 genotypes. Accession labels were color coded according to subspecies and the nodes according to market types

A phylogenetic network was constructed with 6300 SNPs filtered for MAF ≥ 0.05. The comparison of the clusters and market classes showed significant admixture. The reticulation pattern suggests a complex network relationship amongst the 107 accessions plus 6 cultivars – likely reflecting common breeding histories along the diversification paths of these accessions and some degree of hybridization among the accessions (Fig. 3).

Accessions clustered together in five main groups, with some correspondence to subspecies and market class, but with numerous exceptions (Fig. 3). The top right nodes in Fig. 3 consisted predominantly of Valencia type accessions from *fastigiata* subspecies and a few Spanish and mixed types whereas the top left nodes consisted mostly of Spanish type accessions that were classified as *hypogaea* subspecies with only a few *fastigiata* subspecies*,* even though Spanish types are classically *fastigiata* subspecies var. *vulgaris*. The bottom right nodes were predominantly hypogea with a few *fastigiata* whereas the bottom left consisted mostly of *fastigiata* and a few *hypogea* subspecies. The nodes in the mid-section constituted the fifth group with all the commercial cultivars except Tamnut OL 06 [32] (Fig. 3). A Fisher exact test showed significance that the observed clustering pattern based on genotypes corresponds approximately with subspecies and market type grouping, with *p*-values = 5.8 × 10^− 10^ and 1.8 × 10^− 6^ respectively.

### Linkage disequilibrium and minor allele frequency

To study the effect of minor alleles on the nature of LD and the extent of its decay, three different MAF threshold cutoff levels (0.05, 0.1 and 0.2) were tested. Mean LD among all SNPs was calculated across the entire genome over different map distances. The SNPs were pooled over all chromosomes in each sub-genome to compute genome-wide mean LD for each distance bin.

**Fig. 4:**
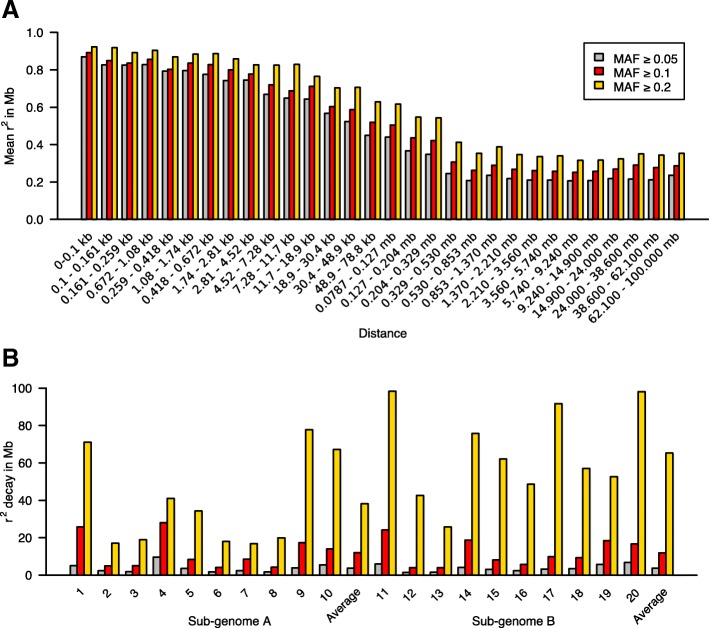
Effect of MAF on the nature of LD and its decay in the mini core collection. **a** Mean LD estimates computed at different map distances across the entire genome for three varying MAF threshold cutoffs. **b** LD decay with distance across the 20 chromosomes measured with varying MAF thresholds for *r*^2^ = half decay distance. LD decay distance is given in mega base pairs

Mean LD estimates generally declined with increasing bin distance and LD was significantly affected by MAF, especially over longer distance bins (> 7.28 kb), where mean LD declines to approximately half its original value (Fig. 4a). For MAF ≥ 0.05, mean LD estimates ranged from *r*^2^ = 0.87 (0–0.1 kb) to *r*^2^ = 0.236 (distance > 62,100 Mb). Mean LD is high (*r*^2^ > 0.82) at short distance bins (< 0.5 kb) and declines with increasing bin distance. It drops to *r*^2^ = 0.44, which is approximately half the original value, at bin distance 78.7 kb – 127 kb (Table 1; Additional file 8: Table S5).

**Table 1:** Mean LD estimates among all SNPs with MAF ≥ 0.05 at different physical distances across the genome

Distance (kb)	*n*	mean *r*^2^	SD *r*^2^	Distance (kb)	*n*	mean *r*^2^	SD *r*^2^
0–0.10	301	0.870	0.268	78.8–127.0	1600	0.440	0.388
0.10–0.16	141	0.827	0.318	127.0–204.0	1903	0.367	0.366
0.16–0.26	225	0.826	0.314	204.0–329.0	3005	0.348	0.352
0.26–0.42	278	0.828	0.321	329.0–530.0	3630	0.245	0.283
0.42–0.67	348	0.793	0.340	530.0–853.0	4385	0.208	0.248
0.67–1.08	416	0.796	0.344	853.0–1370.0	7389	0.236	0.265
1.08–1.74	489	0.776	0.357	1370.0–2210.0	12,202	0.218	0.243
1.74–2.81	427	0.743	0.369	2210.0–3560.0	17,026	0.210	0.236
2.81–4.52	378	0.745	0.358	3560.0–5740.0	22,778	0.210	0.235
4.52–7.28	331	0.669	0.398	5740.0–9240.0	25,869	0.206	0.225
7.28–11.7	331	0.649	0.406	9240.0–14,900.0	31,364	0.207	0.229
11.7–18.9	612	0.644	0.392	14,900.0–24,000.0	38,171	0.219	0.236
18.9–30.4	603	0.568	0.408	24,000.0–38,600.0	45,340	0.216	0.245
30.4–48.9	808	0.524	0.404	38,600.0–621,000	43,255	0.212	0.246
48.9–78.8	1027	0.450	0.386	62,100–100,000	94,337	0.236	0.254

*n* number of *r*^2^ pairwise values*, SD* standard deviation*, kb* 1000 base physical distance

### LD decay with distance

The LD statistic *r*^2^ was used to estimate LD between pairwise comparisons of markers with sliding window size of 50 markers for each of the 20 chromosomes filtered for MAF greater than 0.05, 0.1, and 0.2. LD decay distance increases with MAF, the increase is very significant at MAF ≥ 0.2 (Fig. 4; Additional file 8: Table S6). At MAF ≥ 0.05, LD decays to *r*^2^ = half decay distance at 3.78 Mb. The rate of decay varies for each chromosome. LD persists the longest in chromosome 4 (9.67 Mb) and chromosome 20 (6.75 Mb). The decay distance is shortest in chromosome 12 (1.41 Mb) and chromosome 13 (1.54 Mb). Chromosomes 4 and 14 have high LD, as do chromosomes 10 and 20 (Table 2; Additional file 2: Figure S2; Additional file 3: Figure S3a).

**Table 2:** Average LD decay distance estimated for each chromosome and sub-genome

Chromosome	Minor Allele Frequency		
Sub-genome A	≥ 0.05	≥ 0.1	≥ 0.2
1	5.07	25.82	71.09
2	2.38	4.99	17.14
3	1.80	5.08	18.94
4	9.67	27.99	41.06
5	3.55	8.41	34.32
6	1.79	4.10	17.97
7	2.34	8.49	16.82
8	1.66	4.24	19.97
9	3.93	17.31	77.74
10	5.50	13.96	67.16
Average	3.77	12.04	38.22
Sub-genome B			
11	5.95	24.13	98.31
12	1.41	4.03	42.65
13	1.54	4.03	25.84
14	4.18	18.74	75.79
15	3.09	8.14	62.11
16	2.36	5.70	48.67
17	3.26	9.86	91.70
18	3.51	9.32	57.06
19	5.77	18.49	52.59
20	6.75	16.72	98.13
Average	3.78	11.92	65.29
Genome-wide Average	3.78	11.98	51.75

Values reported for LD decay at *r*^2^ half decay distance in mb

### Linkage disequilibrium and population structure

To study the effect of population structure on the nature and extent of LD, the mean LD and average LD decay distance were estimated separately for each of the two subspecies *fastigiata* and *hypogaea*, using 6300 SNP markers filtered for MAF ≥ 0.05. Subspecies classifications are based on similar studies [30, 31] shown in detail in Additional file 8: Table SA.

On average, mean LD estimates varied significantly between the two subspecies. Estimates are higher in *hypogaea* than in the *fastigiata* subspecies. Unlike the *hypogaea* subspecies, mean LD values are not significantly different between the *fastigiata* subspecies and the estimates from the larger pool of 113 accessions plus cultivars. The decay distance is significantly longer in *hypogaea* subspecies (average decay distance = 13.52 Mb) than in *fastigiata* (average decay distance = 3.41 Mb). There was no significant difference between the extent of decay between *fastigiata* subspecies and the larger collection - average decay distance in the 113 collection = 3.78 Mb (Additional file 3: Figure S3; Additional file 8: Table S7).

### Genome-wide association analysis (GWA)

**Fig. 5:**
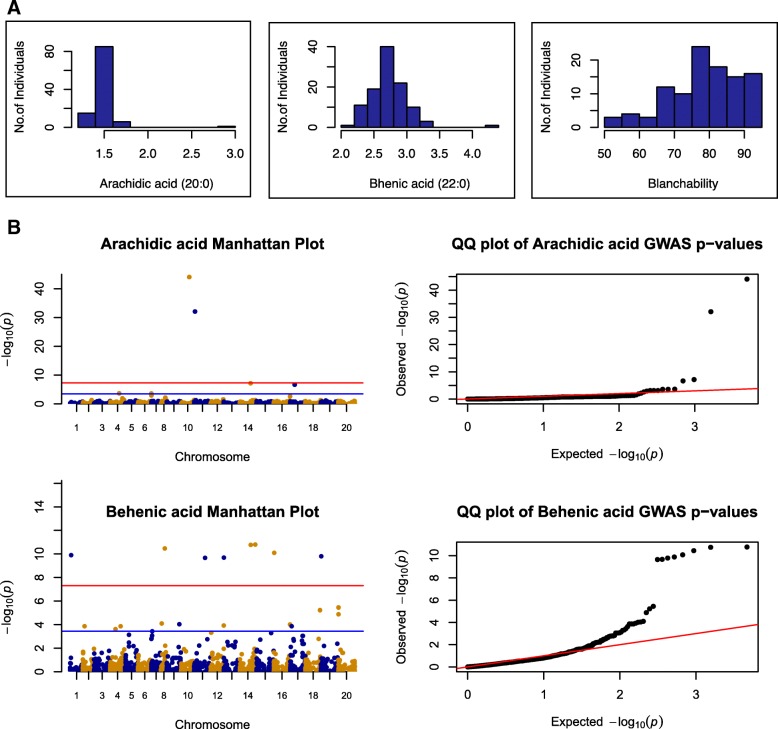
Association results for arachidic and behenic saturated fatty acids. **a** Histogram showing phenotypic distribution of arachidic, behenic fatty acids and blanchability. The x-axis shows BLUP values over three replicates for fatty acids and BLUPs over three locations for blanchability. The y-axis represents the number of individuals. **b** Manhattan and QQ plots for Arachidic and Behenic fatty acids. In the Manhattan plots, the blue horizontal line represents a suggestive line defined at 3.71 × 10^− 4^ and the red line represents an adjusted 5% multiple tests Bonferroni corrected threshold cut off at 1.86 × 10^–5^

The SNP-basedgenotype-phenotype associations for fatty acid composition are displayed in Manhattan plots of –log (*p*-values) and in Q-Q (quantile-quantile) plots of expected (under a Gaussian distribution) versus observed p-values (Fig. 5; Additional file 4: Figure S4; Additional file 5: Figure S5; Additional file 6: Figure S6; Additional file 7: Figure S7). Additionally, the Manhattan and Q-Q plots for total protein, total oil content and blanchability are shown in Additional file 7: Figure S7. The most probable associations detected in the GWA study are listed in Table 3; Additional file 8: Table S8, where, for the largest peaks, only the most significant SNPs are reported.

**Table 3:** Summary of significant associations and predicted candidate genes for arachidic and behenic fatty acids

Trait	Marker	Chr	Pos	*P*-value	Marker *r*^2^	Candidate genes	Gene annotation
Arach-idic	AX-147237808	B01	6,039,768	3.52E-09	0.51845424	Araip.ZQ4WS	Non-lysosomal ceramidase
	AX-147237808	B01	6,039,768	3.52E-09	0.51845424	Araip.9YT86	Non-lysosomal ceramidase
	AX-147255620	B07	37,296,448	4.96E-06	0.316757	Araip.L66QB	Flavin-binding Monooxygenase family protein
	AX-147255620	B07	37,296,448	4.96E-06	0.316757	Araip.L66QB	ATP binding Microtubule motor family protein
	AX-147220134	A04	74,357,243	1.80E-04	0.214561	–	–
Behenic	AX-147247765	B04	90,017,134	1.74E-11	0.38628	Araip.10024352	Palmitoyl protein thioesterase family protein
	AX-147248572	B04	127,788,433	1.64E-11	0.38871	Araip.10024375	Acetylglucos-aminyltransferase familyprotein
	AX-147248572	B04	127,788,433	1.64E-11	0.38871	Araip.10024487	Thioesterase superfamily protein
	AX-147251762	B06	3,275,121	8.25E-11	0.39006	Araip.10017394	Fatty acid desaturase 2
	AX-147259171	B08	122,074,815	5.99E-06	0.23662	–	–
	AX-147209429	A01	9,155,638	1.30E-10	0.3855	Aradu.10033983	Diacylglycerol Acyltransferase family
	AX-147209429	A01	9,155,638	1.30E-10	0.3855	Aradu.10033986	Diacylglycerol Acyltransferase family

*Abbreviations* in the table*: chr* chromosome*, pos* Marker position

Arachidic and behenic saturated fatty acids showed moderately strong associations in this study. Several plausible candidate genes associated with fatty acid metabolism were identified in these regions (Table 3). At the 5% adjusted Bonferroni threshold, eight markers showed significant association with behenic saturated fatty acid, and two showed associations with arachidic fatty acid. The marker AX-147251762; 3,275,121 bp on chromosome 16 (B06) was found to associate with behenic acid with *p*-value of 8.25E-11. This SNP is 356.1 kbp upstream of a fatty acid desaturase 2 gene, Araip.D6HPL (Araip.B06:3631192..3632515). There are 21 other gene models predicted within this region. Additionally, markers AX-147247765; 90,017,134 bp and AX-147248572; 127,788,433 bp on chromosome 14 (B04), had significant associations with respective *p*-values of 1.7E-11 and 1.6E-11. Marker AX-147248572, is 53.67 kbp upstream of Araip.R5W4R (Araip.B04:127839622..127842106), which is predicted as a thioesterase superfamily protein.

The SNP AX-147237808 at 6,039,768 bp on Chromosome 11 (B01) significantly associated with arachidic acid with p-value of 3.52E-09. This marker is 75.96 kbp upstream of Araip.9YT86, a non-lysosomal ceramidase predicted gene. Four other genes are predicted within this 75.96 kbp region. SNP AX-147255620, at 37,296,448 bp on chromosome 17 (B07), is associated with arachidic acid with p-value409E-06. This marker is 66.12 kbp downstream of a flavin-binding monooxygenase family protein (FMO3), Araip.L66QB (Araip.B07:37228337..37230328).

Each of the top SNPs for the remaining weak, non-significant marker- trait associations were examined and additional candidate loci that could explain the observed marker association were identified (Additional file 8: Table S8).

### Blanchability in the U.S. peanut mini core

Multiple tests were carried out for blanchability using both GLM-PCA and MLM-PCA + Kinship models. Two markers consistently showed strong, though not significant associations at the adjusted Bonferroni corrected cutoff threshold, for all the tests. Marker AX-147230936 at 34,653,638 bp on chromosome 8 (A08) showed association with a *P*-value of 2.14E-04 and marker AX-147253931 at 131,362,404 bp on chromosome B06 with a *P*-value of 2.74 E-04 (Additional file 7: Figure S7; Additional file 8: Table S8).

The analysis was repeated using the GAPIT package in R version 3.3.2 using MLM with PCA and Kinship. And again, SNP AX-147253931 at 131,362,404 bp on chromosome 16 (B06) with a *P*-value of 1.56E-04 and SNP AX-147247686 at 76,220,065 bp on chromosome 14 (B04) with a *P*-value of 3.99E-04 showed strong, though not significant association (Additional file 8: Table S8).

We found the genomic region spanning 130,111 kb to 131,362 kb on chromosome 16 (B06) to consistently associate with blanchability despite the lack of statistical significance. This genomic region has 75 candidate genes, three of which show elevated expression in the seed pericarp: Araip.LS9BW and Araip.Q8ZS3, which are chalcone synthases, and Araip.J6A49, which is an ATP binding protein (Additional file 8: Table S8).

## Discussion

### Evaluation of SNP characteristics

Some markers consistently exhibit unusually high levels of heterozygosity across many accessions that otherwise appear homogenous. The excessively heterozygous markers could be due to the probes detecting homeologs and failing to distinguish between the two highly similar sub-genome sequences. Homeologous sequences which are a result of chromosome duplication events in meiosis I and polyploidy, generate interfering signals to DNA bases being assayed which makes it hard to score SNP markers in peanut and other polyploids [9].

Nine mini core accessions have high levels of heterozygosity, ranging from 10 to 28%, and several other accessions showed moderate, but greater than expected levels of heterozygosity – these could be a result of recent hybridizations - even though peanut is putatively highly self-pollinating. Since the purified mini core population was not generated via single seed descent method [5], the individuals chosen for seed increase could have been highly heterozygous from the start, and the heterozygosity persisted during seed increase.

Outcrossing during seed increase could also explain some of the high heterozygosity levels reported. Ambiguous SNP calls mostly occurred in particular regions of the genome in one or a few accessions, often interspersed with tetrasomic calls (tetrasomic regions occur where sequence exchange and homogenization has occurred between the subgenomes). These putatively-tetrasomic regions showed little or no diversity, and therefore were excluded from the set of 13,527 informative markers analyzed.

Almost all the highly heterozygous markers had negative inbreeding coefficients, while most SNPs had an inbreeding coefficient of one. The inbreeding coefficient (**f**), measures the probability that two alleles at any locus in an individual are identical by descent from the common ancestor(s) of the two parents. These results demonstrate that the mini core accessions have little or no evidence of inbreeding except for a few loci where **f** was less than one.

### Genetic diversity estimates

Tajima’s D distinguishes between randomly changing loci and those evolving non-randomly as a result of directional selection, introgression, genetic bottleneck and/or drift [33]. Generally, a negative value for Tajima’s D is indicative of an excess of rare alleles within a collection. The observed Tajima’s D value of − 0.00252 shows that the mini core collection is evolving mostly neutrally although filtering for less heterozygous markers could have left fewer than expected rare alleles in the collection. For the mini core collection, such a phenomenon could probably have been due to the selection for diversity in the U.S. peanut mini-core and may also reflect population size expansion after a bottleneck – such as following the origin of tetraploid peanut. A large proportion of the markers had minor alleles with frequency less than 5%. Small populations tend to have less allelic diversity compared to much larger populations [34]. Also, although the mini core was selected to maximize the genetic diversity in the peanut core and germplasm collection, we found a high amount of genetic similarity among the accessions which is unsurprising since peanut has long been known to have low genetic diversity to start with. Better SNP calling algorithms with the ability to predict SNP positions and distinguish true homologous SNPs from homeologous ones could improve marker behavior and consequently conclusions drawn from SNP-based studies in peanut.

### Population structure and phylogenetic analysis

Understanding population structure is useful for effectively utilizing genotypes for breeding purposes. FastStructure is a variational Bayesian framework for posterior inference that assigns individuals in a sample to a subpopulation, or jointly to two or more subpopulations for genotypes with admixed sets of alleles at their loci. Structure analysis identified 4 to 5 subgroups, similar to other studies [30, 31].

Results also show high levels of admixture, which supports hybridization or outcrossing among the individuals. Although it has been shown that minor alleles affect population structure this is not apparent in the U.S. peanut mini core [35]. This could be due to the small population size under study (only 107 accessions used in the structure analysis).

The evolutionary relationship(s) between nucleotide sequences, genes and/or species is revealed through a phylogenetic analysis and is often displayed in the form of a bifurcating phylogenetic tree. However, in cases where hybridization, recombination, gene duplication or horizontal gene transfer is believed to have occurred, a bifurcating tree structure is insufficient in displaying such reticulate relationships. The complex clustering pattern shown in the SplitsTree4 graph is consistent with the high levels of admixture observed in the structure analysis. In both the structure and phylogenetic network analyses, the clustering pattern is not consistently explained by market type nor and subspecies classifications, although a Fisher exact test showed significance in correspondence. This is similar to findings in previous studies using SSR makers [30, 31, 36]. The numerous exceptions may indicate that traits associated with market type are determined by small genomic regions, so that the phylogenetic signal from those regions is swamped by other regions. It may also indicate that market type traits are complex, subjective, and difficult to measure even by experts in the field.

Another probable reason for the incoherent clustering pattern of the genotypes by market class and sub species groupings could be that both classifications were inconsistently scored lending strong genotypic evidence to the need for reclassification as previously observed [37]. Classifying accessions according to market type is highly subjective and the traits are difficult to accurately score in the field. In any case, market type may not be well predicted by genotype and thus phylogenetic clustering is only loosely associated with market type.

### Linkage disequilibrium in the U.S. peanut mini core

LD is quantified by comparing haplotype frequencies observed in the data to their expected frequencies based on independence. In the U.S. mini core collection, mean LD estimates generally declined with increasing bin distance as expected. Mean LD dropped from 0.87 to half *r*^2^ of 0.44 at 78.7–127 kb. LD estimates were significantly affected by MAF, especially over longer bin distances. This is contrary to findings in maize where MAF was shown to significantly affect mean *r*^2^ estimates, especially at short distances between 0 to 10 kb and the effect was not so pronounced over longer bin distances [38].

Minor alleles shift *r*^2^ values down, leading to the underestimation of LD means and LD decay distances. This is consistent with findings that showed that rare SNPs tend to have lower pairwise values compared to more common SNPs [39]. The dramatic difference in LD estimates with varying MAF thresholds suggests that genome-wide estimates of LD based on relatively few samples are very sensitive to MAF compared to relatively larger number of samples with over 21,000 SNPs or LD estimates in short sequence stretches [40, 41]. It is widely recommended to filter SNP data for MAF. The most commonly used threshold of MAF > 0.05 gives moderate and more representative *r*^2^ mean values and LD decay distances compared to all the other thresholds investigated.

Average LD estimates persisted over long distances, decaying to *r*^2^ = half decay distance at 3.78 Mb in the collection. Contributing factors could be that cultivated peanut is a self-fertilizing species (resulting in slower breakup of LD blocks), and likely went through an extreme bottleneck at the time of tetraploidization. This means that it is expected to have a mostly homozygous genetic background with a relatively low number of detectable recombination events between loci, thus the observed low rates of LD decay across the genome [42, 43]. In the *hypogaea* subspecies, LD decays over a significantly longer distance compared to the decay distance in *fastigiata* subspecies.

Our results show little or no difference in LD decay distance between *fastigiata* subspecies and the entire collection, which is contrary to results from a study using 392 SSR markers that reported LD to have persisted over 10 cM in the entire mini core collection and 20 cM in *fastigiata* subspecies [31]. However, LD has also been shown to be higher in small sample sizes, with the trend being more noticeable in LD measured across marker intervals greater than 5 kb [38]. Therefore, we suggest caution when interpreting these results, especially the marked disparity in LD decay distance between the two subspecies *fastigiata* and *hypogaea* which was investigated using a subset of 59 and 67 accessions, respectively. The extent of LD is highly heterogenous across chromosome regions – generally long in genic regions and short in intergenic regions. A low and non-uniform SNP density across each chromosome (especially after filtering for MAF > 5%), hampers the ability to draw conclusions on the variable patterns of fine-scale LD across the genome except for general trends since SNP density is not the same in genic vs intergenic regions.

Altogether, these results show that LD persists over a long distance in the U.S. mini core peanut collection and that the nature of LD and the extent of its decay are affected by minor alleles as well as population structure. These large blocks of persistent LD with a low decay rate make it hard to achieve high resolutions for fine mapping, or for identifying marker-trait associations. GWA relies on markers proximal to the causative loci being in high LD with the QTL but this should quickly drop for those markers further from the QTL otherwise mapping resolution is limited. Low mapping resolution can be overcome by genotyping at a higher marker density or by using collections that have experienced sufficient rounds of recombination within the desired region to breakdown persistent LD blocks.

### LD based association mapping

The mini core collection was phenotyped for several biochemical traits but characteristics of the collection and of the measured traits resulted in generally insignificant association values except for arachidic and behenic fatty acid composition, which showed significant associations. Although present in very small quantities in peanut, behenic and arachidic fatty acids have been shown to raise cholesterol levels in humans [44].

Based on an adjusted 5% Bonferroni corrected cutoff threshold of 1.86 × 10^− 5^, two markers appear to associate significantly with arachidic acid and eight with behenic acid. These markers offer good targets for further validation and future studies based on their proximity to candidate genes implicated in some form of fatty acid metabolism. The SNP on B01 is near two predicted non-lysosomal ceramidase proteins, Araip.ZQ4WS and Araip.9YT86. Ceramidase has been shown to catalyze the hydrolysis of ceramide to sphingosine and fatty acid, and possibly also the reverse reaction [45]. The SNP on chromosome B07, is 66.12 kbp downstream of a flavin-bindingmono-oxygenase family protein which has been linked to polyunsaturated fatty acid metabolism and lipid homeostasis [46].

For behenic acid, the SNP on chromosome B06 was found to be 356.1 kbp upstream of Araip.D6HPL predicted as a fatty acid desaturase 2 gene (FAD2) with GO terms for lipid metabolic process and oxidation-reduction process. FAD2 enzymes catalyze the oxidation of oleic acid to linoleic acid [47–49] thus increasing rancidity and off flavors. Functional mutations in the *ah*FAD2 genes, was shown to control the conversion of oleic acid to linoleic acid during seed development and thus resulted in the targeted breeding of “Hi-Oleic” peanuts with an improved shelf-life [50, 51]. There are 21 other gene models predicted within this 356.1 kbp region.

Two other SNPs on chromosome B04 are in proximity to two gene models with the predicted function of a palmitoyl protein thioesterase family. Palmitoyl protein thioesterases catalyze the removal of thioester-linked fatty acyl groups like palmitates from modified cysteine residues [52]. These genes, Araip.2TH5J and Araip.R5W4R, are associated with GO Terms; GO:0008474 for palmitoyl-(protein) hydrolase activity and GO:0002084 for protein depalmitoylation. Lastly, the marker on A01 was found proximal to Aradu.6I2MF and Aradu.UR9Q8, predicted as diacylglycerol acyltransferase protein family. Diacylglycerol acyltransferase 2 has been shown to link glucose utilization to fatty acid oxidation. It has been shown to specifically facilitate the channeling of de novo synthesized fatty acids into a rapidly mobilized pool of triacylglycerol [53]. These genes are associated with GO:0004144 for Diacylglycerol O-acyltransferase activity.

Associations for blanchability were not significant at the applied adjusted Bonferroni threshold but the region on chromosome B06 had plausible candidate genes with strong expression in the seed pericarp and require additional evaluation. Chalcone synthase is the first committed enzyme in the flavonoid biosynthesis pathway which among other things is associated with pigmentation and plant defense mechanisms against stress [54]. In peanut, blanchability is the ease with which the seed coat is completely removed from the seed prior to processing into various confectionery products. Most of the reported candidate genes show tissue expression in the seed and pericarp except for a few, like Aradu.UR9Q8, which is mostly expressed in the peg, vegetative shoot tip and mainstem leaf [55]. These results demonstrate the potential for GWA to discover candidate genes and reliable markers associated with important traits for peanut, despite using relatively few accessions.

In addition to these traits, several other fatty acid components, total oil and total protein were analyzed for associations. The lack of significance in association for many of the traits could be due to several factors, including multigenic or complex control of the traits, small sample size, structure, and genetic similarity within the mini core accessions. The power of GWA to detect a true association between a SNP and a trait of interest has been shown to be limited by both the effect size and frequency of occurrence of the allelic variant(s) explaining the trait within the population [42, 56, 57]. A small population size might therefore lead to the detection of significant GWA signals for traits, if such are, under the control of a single gene or a small group of genes with large effect sizes. However, this is still highly dependent on the characteristics of the population like the nature and extent of LD, kinship and structure [42, 58, 59].

From structure results, we were able to identify four to five sub-populations, but GWA results were optimized at two to four PCA levels depending on the trait. This is not atypical as PCA estimates genetic background derived from a set of independent genetic markers and uses these to control for population stratification whereas methods like FastStructure use a set of unlinked markers to estimate ancestry probabilities for each individual and thus provides more information about the level of stratification within a collection of individuals [60]. Even after controlling for structure, there are still other confounding factors that remain unaccounted for, as shown in deviations from the main diagonal in the QQ-plots. Unrecognized population structure or admixture confounds associations between genotypes and phenotypes leading to false results [23].

Unlike with Arabidopsis and other highly self-fertilizing species, where a small collection number was sufficient for GWA to detect strong associations [42, 58, 59], our collective results show that this is not plausible with peanut due in part to genome complexities coupled with a close kinship and relatedness amongst accessions and cultivars. A similar study using 81 SSR markers reported no associations except for the well characterized ahFAD2 markers and an association for linoleic acid [30]. It requires a relatively large and carefully chosen collection of accessions for GWAS to detect meaningful associations in peanut, even for qualitatively inherited traits with large effects, in spite of its highly self-fertilizing nature.

## Conclusions

This study offers insights into peanut diversity and provides valuable information to peanut breeders and geneticists toward variety improvement. The study provides information about the relatively high degree of structure in the U.S. peanut mini core collection and suggests that structure will likely be a challenge for most association studies in peanut – as will the generally-high LD, and complications due to paralogy of markers in this tetraploid species. Nevertheless, this study did identify some marker-trait associations, even considering the challenges of this collection and species. We found LD to persist over long distances in the collection, decaying to *r*^2^ = half decay distance at 3.78 Mb. The nature of LD and the extent of its decay varied widely across the genome and is affected by both minor allele frequency and population structure. Structure within the collection is best explained when separated into four or five groups (K = 4 and K = 5) with high admixture proportions. In general, our findings show that the U.S. peanut mini core collection may not be well suited for GWAS due to its limited population size, structure/genetic similarity, and long LD blocks that limit the resolution of association mapping. We identified candidate loci for traits analyzed and suggest follow-up studies. Work is ongoing to genotype the larger core collection of approximately 831 accessions to facilitate the discovery of reliable markers associated with traits of interests and facilitate the discovery of new genes or allelic variants controlling these traits.

## Methods

### Phenotypic evaluation in the U.S. peanut mini core

Seeds were obtained from the USDA Plant Genetic Resources Conservation Unit (PGRCU) in Griffin, Georgia and planted at the University of Florida Plant Science Research station in Citra, Florida, using the standard planting procedures at the PGRCU for peanut. In brief, each accession was planted in a two-row plot 3 m in length with 75 cm row spacing. Each plot was spaced 3 m apart in the planting direction with a 1.5 m inter-row spacing to minimize cross contamination. Seeds were planted at a density of 50 seeds/row at a depth of 3.5 cm. The experiment was planted using an augmented randomized block design with three blocks over three growing seasons (2013, 2014 and 2015). The 107 mini-core lines were replicated once in each block, along with six commercial standards in each block. These included: Bailey [61], Florida Fancy (PVP 200800231 Sep 2012), Jupiter [62], Red River [63], Tamrun OL11 [64] and Tamnut OL 06 [32].

### Biochemical analysis

Three seeds of harvested mature peanuts were flash frozen with liquid nitrogen, ground to a fine powder and stored at − 20 °C prior to analysis. Biochemical data were collected on total protein content, total oil content, and fatty acid composition. Protein content in seed was calculated using total nitrogen detection via Kjeldahl digestion, and the standard conversion factor of 5.46 was used for raw protein content calculation. Total oil was calculated using an extraction procedure from Jean Thomas (unpublished). In brief, approximately 2 ml of 50–50 hexane tert-butyl ether was added to 0.2 g of ground tissue in a tube. The tube was vortexed, capped and placed under a fume hood for 10 h. The tube was centrifuged and the supernatant carefully transferred into a pre-weighed 16 × 125 mm tube (call this weight **a**). This process was repeated three times to yield approximately 6 ml of total supernatant in the pre-weighed tube. The tube was then placed in an evaporating chamber in a water bath heated at 40 °C and nitrogen gas was passed into the chamber to purify the oil from the hexane tert-butyl ether extractant. The tube, now containing purified oil, was re-weighed (call this weight **b**). The weight of the pre-weighed tube was subtracted from this weight to give the weight of the extracted oil. The percent oil composition was calculated as: Y = (b-a) / (0.2*3).

To determine fatty acid composition, one drop of the extracted oil (approximately 0.025 g) was dissolved in 200 μL of hexane. 200 μL of an esterification mixture containing one-part sodium methoxide, four parts petroleum ether, and two parts ethyl ether was added to the vial, and vortexed. An additional 600 μL of hexane was added to the vial, vortexed, and allowed to sit for 30 min, at room temperature, prior to analysis. An Agilent 7890 gas chromatographer unit equipped with a flame ionization detector (FID) was used for fatty acid determination. A 15 m Agilent/J&W DB-225narrow-bore column (0.25 mm) with a 60:1 split inset was set to an internal temperature of 280 °C. A 1 μL injection volume was used and the carrier gas, helium, was set at a flow rate of 1 mL/minute. The detector temperature was set to 300 °C and total run time was set to 17 min per sample. The retention time in minutes for the fatty acids are as follows: palmitic (1.619), stearic (2.465), oleic (2.638), linoleic (2.878), arachidic (3.500), gadoleic (4.154), behenic (6.419) and lignoceric (9.328). The resulting peak heights were recorded, and the height of individual peaks was divided by the combined height of all peaks to calculate percentage of total oil for each fatty acid component.

Blanchability in peanut is the ability to completely remove the seed coat from the seed. It was evaluated in the peanut mini core in three environments (Australia 2013, U.S.A 2013 and Australia 2014) as described previously [65]. Blanching was found to be a highly heritable trait with variation mainly explained by genotypic variance rather environmental variance [65]. An overall prediction of the genotypes across the three locations was used to generate best linear unbiased predictors (BLUPs) for each genotype.

### Genotyping, SNP performance and quality

All seeds for this study were ordered from the purified peanut mini core collection [5] maintained at USDA-ARS Plant Genetic Resources Conservation Unit in Griffin, GA. DNA was extracted directly from a single seed per accession using an E.Z.N.A. Omega Bio-Tek kit (Doraville, GA). A single seed for each genotype (accession) was utilized to extract DNA. A small chip (~ 75 mg) distal from the embryo of each genotype, was cut using a razor blade placed in a 2 mL tube along with two tungsten carbide beads and P1 buffer from the kit. DNA was extracted from the chip following the instructions from the E.Z.N.A. DNA extraction kit.

Of the 58,000 SNP positions present in the Affymetrix fixed array [13, 14], all SNP positions were evaluated and 13,527 SNPs were selected for use in this study on the basis of interpretability (ability to score alleles as coming from the A or B subgenomes) and for polymorphism relative to the U.S. peanut mini core plus six select commercial varieties.

### Genetic diversity and haplotype blocks

SNP genotype data was used to study genetic diversity and the genetic relationship among individuals of the mini core collection. Allele frequencies, major and minor gamete frequencies were calculated using the software TASSEL version 5.2.39 using default settings [66].

The polymorphic information content (PIC), heterozygosity, within-population inbreeding coefficient and gene diversity was calculated using the software PowerMarker version 3.25 using the default settings [67].

The average pairwise divergence among genotypes, which represents the nucleotide diversity per bp, π (pi) and the expected number of polymorphic sites per nucleotide, θ (theta), were estimated using the software program TASSEL v5.2.39 using the default settings. The normalized measure of difference between the observed (π) and expected (θ) nucleotide diversity, Tajima’s D, was also computed. Haplotypes were determined using options “-dog --blocks no-pheno-req” in PLINK v1.90b4.4 [68]. The maximum size of blocks was set at the default level of 2 Mb.

### Population structure

Population structure was determined using the software program FastStructure, version 1.0 and the appropriate number of model components that explain structure in the dataset was determined by running a python script, chooseK.py [28]. Admixture proportions were visualized in R statistical software program version 3.3.2 using the R package, Pophelper version 2.2.3 [69]. A phylogenetic network was constructed using the SplitsTree4 software [70]. Market type, botanical variety and subspecies classifications were obtained from GRIN-Global (https://npgsweb.ars-grin.gov) and previous publications [30, 31, 46].

### Linkage disequilibrium and decay

SNP markers were filtered for a minimum count of 100 known alleles and minor allele frequency (MAF) of 0.05, 0.1, and 0.2. LD analysis was performed for each chromosome, by computing *r*^2^ values for all pairwise marker comparisons using a sliding window size of 50 markers around the current site, in TASSEL v5.2.39. Marker positions were then used to investigate LD decay along each chromosome and across the entire genome. Background LD was estimated as the 90th percentile of the *r*^2^ value of marker-pairs on different chromosomes. LD decay distance was determined by fitting a non -linear model using the Hill and Weir method, later modified by Remington et al., with *r*^2^ threshold set at 0.2 and *r*^2^ = half decay distance. To estimate the effect of population structure on LD decay, LD decay within each subspecies was analyzed.

### Genome-wide association analysis

Genome-wide association (GWA) was performed using a weighted mixed linear model (MLM) at optimum compression level and variance components were estimated once using P3D in TASSEL version 5.2.33 and in R using the R package GAPIT [71, 72]. A kinship matrix was generated in TASSEL using Centered-Identity by State (Centered IBS) with two maximum alleles using 6300 SNP markers filtered for MAF ≥ 0.05. Population structure was accounted for using Principal components calculated in TASSEL and GWA was run using the model: Trait = BLUPs + PC + Kinship + marker.

We applied a whole-genome significance cutoff based on an adjusted Bonferroni test threshold at 1.86 × 10^− 5^ and a suggestive line defined at 3.71 × 10^− 4^ following a modified Bonferroni correction method described by Li et al. 2012 [73]. Candidate genes were predicted using genomic intervals of two non-significant SNPs flanking a significant SNP associated with the trait of interest. The interval was queried against the peanut base genome browser https://peanutbase.org [8] to identify genes that have known functions associated with the trait.

## Additional files

Additional file 1: **Figure S1.** Population structure in the mini core for K = 2 to K = 7. The Y-axis represents the probability of assigning an accession to a group and the X-axis accession names. (a) Different K groups ordered according to subspecies. (b) Groups ordered according to botanical variety. (DOCX 9720 kb)

Additional file 2: **Figure S2.** LD decay using Loess fit (black) and non-linear fit (blue) for each of the 20 chromosomes. R^2^ values are plotted on the Y-axis against physical distance in base pairs on the X-axis. Heatmaps represent the density of r^2^ across distance. (DOCX 8289 kb)

Additional file 3: **Figure S3.** LD decay pattern along each chromosome and across each sub- species. **(a**) Summarized LD decay with distance for each chromosome and a genome-wide average for each of the two peanut sub-genomes. (**b**) LD decay in each of the two sub-species at different physical distances. (DOCX 1186 kb)

Additional file 4: **Figure S4.** Histograms showing the phenotypic distribution of seed composition and quality traits in the mini core collection. The x-axis shows BLUP values over three replicates and the y-axis represents the number of individuals with the respective values. (DOCX 276 kb)

Additional file 5: **Figure S5.** Manhattan and QQ – plots for Saturated Fatty acid components. (DOCX 449 kb)

Additional file 6: **Figure S6.** Manhattan and QQ – plots for Unsaturated Fatty acid components. (DOCX 427 kb)

Additional file 7: **Figure S7.** Manhattan and QQ – plots for Oleic-linoleic ratio, total oil, total protein content and Blanchability. (DOCX 377 kb)

Additional file 8: **Tables S1. – S8.** Sheets in the Table contain details of the accessions used in the study, overall SNP summary, heterozygosity among accessions, SNP distribution, nucleotide diversity, haplotypes, Mean LD and LD decay estimates as well as detailed GWA results for all the traits evaluated. (XLSX 1396 kb)

## Data Availability

The datasets generated and/or analyzed during this study are available in supplementary files and through PeanutBase using the following links for trait and genotype data respectively: https://peanutbase.org/data/public/Arachis_hypogaea/minicore_Dezern.trt.JWYM https://peanutbase.org/data/public/Arachis_hypogaea/aradu1_araip1.gnm1.div.2B6N

## Abbreviations

GWA: Genome wide association
ICRISAT: The International Crops Research Institute for the Semi-Arid Tropics
LD: Linkage disequilibrium
LDL: Low Density Lipoprotein
QTL: Quantitative trait loci
