# Supplementary material for: Evaluation of linkage disequilibrium, population structure, and genetic diversity in the U.S. peanut mini core collection
Source: BMC Genomics. 2019 Jun 11;20:481. doi: 10.1186/s12864-019-5824-9 (PMC6558826; doi:10.1186/s12864-019-5824-9)
Supplement: Supplementary file 1 — Figure S1. Population structure in the mini core for K = 2 to K = 7. The Y-axis represents the probability of assigning an accession to a group and the X-axis accession names. (a) Different K groups ordered according to subspecies. (b) Groups ordered according to botanical variety. (DOCX 9720 kb) [file 12864_2019_5824_MOESM1_ESM.docx]

**Figure S1: Population Structure in the mini core for K = 2 to K = 7**

**Top:** Population structure for K = 2 to 7. (**A**) Different K groups ordered according subspecies. (**B**) Different K groups ordered according to Botanical Variety.
