# Supplementary material for: Evaluation of linkage disequilibrium, population structure, and genetic diversity in the U.S. peanut mini core collection
Source: BMC Genomics. 2019 Jun 11;20:481. doi: 10.1186/s12864-019-5824-9 (PMC6558826; doi:10.1186/s12864-019-5824-9)
Supplement: Supplementary file 2 — Figure S2. LD decay using Loess fit (black) and non-linear fit (blue) for each of the 20 chromosomes. R2 values are plotted on the Y-axis against physical distance in base pairs on the X-axis. Heatmaps represent the density of r2 across distance. (DOCX 8289 kb) [file 12864_2019_5824_MOESM2_ESM.docx]

**Figure S2: LD decay using Loess fit (black) and non-linear fit (blue) for each chromosome**

LD decay plots for each of the peanut chromosomes 1 through 20. Heat maps represent density of r^2^ correlation points by physical distance in base pairs. Black line represents a loess curve function and the Blue a non-linear fit using the Hill and Weir (1988) method. The dashed horizontal line represents the r^2^ = 0.2 decay baseline.
