# Supplementary material for: Evaluation of linkage disequilibrium, population structure, and genetic diversity in the U.S. peanut mini core collection
Source: BMC Genomics. 2019 Jun 11;20:481. doi: 10.1186/s12864-019-5824-9 (PMC6558826; doi:10.1186/s12864-019-5824-9)
Supplement: Supplementary file 3 — Figure S3. LD decay pattern along each chromosome and across each sub- species. (a) Summarized LD decay with distance for each chromosome and a genome-wide average for each of the two peanut sub-genomes. (b) LD decay in each of the two sub-species at different physical distances. (DOCX 1186 kb) [file 12864_2019_5824_MOESM3_ESM.docx]

**Figure S3: LD decay pattern along each chromosome and across each sub- species**

(**A**) Summarized LD decay with distance for each chromosome and a genome-wide average for each of the two peanut sub-genomes. (**B**) LD decay in each of the two sub-species at different physical distances.
