# Supplementary material for: Evaluation of linkage disequilibrium, population structure, and genetic diversity in the U.S. peanut mini core collection
Source: BMC Genomics. 2019 Jun 11;20:481. doi: 10.1186/s12864-019-5824-9 (PMC6558826; doi:10.1186/s12864-019-5824-9)
Supplement: Supplementary file 4 — Figure S4. Histograms showing the phenotypic distribution of seed composition and quality traits in the mini core collection. The x-axis shows BLUP values over three replicates and the y-axis represents the number of individuals with the respective values. (DOCX 276 kb) [file 12864_2019_5824_MOESM4_ESM.docx]

**Figure S4: Histograms showing the phenotypic distribution of seed quality traits in the mini core collection.**

The x-axis shows BLUP values over three replicates and the y-axis represents the number of individuals with the respective values.
