# Supplementary material for: Evaluation of linkage disequilibrium, population structure, and genetic diversity in the U.S. peanut mini core collection
Source: BMC Genomics. 2019 Jun 11;20:481. doi: 10.1186/s12864-019-5824-9 (PMC6558826; doi:10.1186/s12864-019-5824-9)
Supplement: Supplementary file 5 — Figure S5. Manhattan and QQ – plots for Saturated Fatty acid components. (DOCX 449 kb) [file 12864_2019_5824_MOESM5_ESM.docx]

**Figure S5: Manhattan and QQ – plots for Saturated Fatty acid components.**
