# Supplementary material for: Evaluation of linkage disequilibrium, population structure, and genetic diversity in the U.S. peanut mini core collection
Source: BMC Genomics. 2019 Jun 11;20:481. doi: 10.1186/s12864-019-5824-9 (PMC6558826; doi:10.1186/s12864-019-5824-9)
Supplement: Supplementary file 6 — Figure S6. Manhattan and QQ – plots for Unsaturated Fatty acid components. (DOCX 427 kb) [file 12864_2019_5824_MOESM6_ESM.docx]

**Figure S6: Manhattan and QQ – plots for Unsaturated Fatty acid components.**
