# Supplementary material for: Evaluation of linkage disequilibrium, population structure, and genetic diversity in the U.S. peanut mini core collection
Source: BMC Genomics. 2019 Jun 11;20:481. doi: 10.1186/s12864-019-5824-9 (PMC6558826; doi:10.1186/s12864-019-5824-9)
Supplement: Supplementary file 7 — Figure S7. Manhattan and QQ – plots for Oleic-linoleic ratio, total oil, total protein content and Blanchability. (DOCX 377 kb) [file 12864_2019_5824_MOESM7_ESM.docx]

**Figure S7: Manhattan and QQ – plots for Oleic-linoleic ratio, total oil, total protein content and Blanchability.**
